# Accuracy and reliability of novel semi-automated two-dimensional layer specific speckle tracking software for quantifying left ventricular volumes and function

**DOI:** 10.1371/journal.pone.0221204

**Published:** 2019-08-30

**Authors:** Tetsuji Kitano, Yosuke Nabeshima, Yasuhiko Abe, Yutaka Otsuji, Masaaki Takeuchi

**Affiliations:** 1 Second Department of Internal Medicine, University of Occupational and Environmental Health, School of Medicine, Kitakyushu, Japan; 2 Canon Medical Systems, Otawara, Japan; 3 Department of Laboratory and Transfusion Medicine, University of Occupational and Environmental Health Hospital, Kitakyushu, Japan; Northwestern University Feinberg School of Medicine, UNITED STATES

## Abstract

**Purpose:**

To determine whether the semi-automated two-dimensional echocardiography (2DE) layer strain software, compared to cardiac magnetic resonance (CMR), is reliable for left ventricular (LV) volume quantification.

**Methods and results:**

We retrospectively selected 84 patients who underwent CMR and 2DE on the same day. Novel 2DE layer strain software automatically provides LV contour in 3 myocardial layers and performs layer specific speckle tracking analysis, which calculates LV volumes, ejection fraction (LVEF), and global longitudinal strain (GLS) in each layer. These values were compared with reference values from CMR disk-area summation and feature tracking methods. Coverage probability (CP) was determined using predefined cut-off values and absolute differences in LV volumes of 30 mL, those in LVEF of 10%, and those in GLS of 4%. The software did not work in 3 patients (feasibility: 96%). Different layers resulted in different degrees of under- or over-estimation of LV volumes. Epicardial tracking significantly overestimated the LV volumes and significantly underestimated LVEF and GLS. Mid-myocardial tracking had less underestimation of LV volumes and equivalent CP values of LVEF (0.77 vs. 0.75 using the disk-area summation method and 0.90 vs. 0.94 using the feature tracking method) and GLS (0.95 vs. 0.92) compared with endocardial tracking. The new software showed excellent reproducibility, especially endocardial and mid-myocardial tracking.

**Conclusions:**

Mid-myocardial tracking with the novel 2DE strain software provided less bias of LV volumes with high CP values of LVEF and GLS, which suggests that mid-myocardial layer speckle tracking analysis approximates CMR derived LV functional parameters.

## Introduction

Global and regional strain analysis with echocardiography using the speckle tracking software is gaining popularity for the investigation of cardiac chamber mechanics [1]. Although speckle tracking analysis using three-dimensional echocardiography (3DE) is theoretically more accurate and reliable for strain measurements than that using two-dimensional echocardiography (2DE), there have been an increasing number of publications on the use of 2DE strain for the diagnosis of latent ventricular dysfunction [2, 3] and prediction of future outcomes [4, 5]. Continuous efforts from the standardization task force of 2DE longitudinal strain of the American Society of Echocardiography and the European Association of Cardiovascular Imaging make inter-vendor variability of global longitudinal strain (GLS) lower and, thus, facilitate the routine adoption of GLS in daily clinical practice. However, inter-institutional and inter-observer measurement variability still exists predominantly due to variations of manual cardiac chamber border tracing between each institution and each examiner. These results recommend that the same examiner should perform strain analysis in the longitudinal study, and core laboratory should be established to reduce the sample size that needs statistically significant differences in multicenter cross-sectional studies.

Application of fully automated software is one potential solution for this limitation. The automated contour trace (ACT) method is a semi-automated approach to perform left ventricular (LV) layer speckle tracking analysis after clicking three anatomical landmarks on the apical long axis-views. A recently developed prototype software has a potential to further reduce observer variability of LV border determination, resulting in the reduction of measurement variabilities.

Accordingly, the aim of this study was to investigate the reliability and accuracy of the novel 2DE semi-automated layer strain software, compared to those of cardiac magnetic resonance (CMR), for the quantification of LV mechanics.

## Materials and methods

### Study population

We retrospectively selected patients who had undergone clinically indicated CMR and who were also willing to undergo 2DE examination on the same day from June 2017 to May 2018 at the University of Occupational and Environmental Health Hospital. Because patients were selected solely based on the clinical indication of CMR, we did not exclude any patients due to poor 2DE image quality. This study was approved by the ethics committee in the University of Occupational and Environmental Health. Approval number is H30-033. As this was a retrospective study, the Institutional Review Board waived the requirement for informed consent.

### 2D echocardiography

Apical 4-chamber, 2-chamber, and long-axis views with three consecutive cardiac cycle were acquired using the Aplio 300 ultrasound system equipped with a PST-30BT transducer (Canon Medical Systems, Otawara, Japan) before the CMR examination. The receiving frequency was set at 3.0 MHz in the tissue harmonic imaging mode. Imaging data were stored on the hard-disk for off-line analysis. The image quality was assessed from the three apical views and classified into 3 grades (good: 0–2 segments were poorly visible, fair: 3–5 segments were poorly visible, and poor: >5 segments were poorly visible) in the LV 18-segment model according to a previous study [6].

### Cardiac magnetic resonance

The CMR examination was performed using a 3T scanner (Discovery MR750W, GE Healthcare, Milwaukee, USA) with a phased-array cardiovascular coil. In each patient, retrospective electrocardiography-gated localizing spin-echo sequences were used to identify the long axis of the heart. Steady state free precession (SSFP) dynamic gradient-echo cine loops were acquired using retrospective electrocardiographic gating and parallel imaging techniques at 10–15 seconds breath-holds, with the following general parameters: 8-mm imaging plane slice thickness, 40×40 cm field of view, 200×160 scan matrix, 50° flip angle, 3.8/1.7 ms repetition/echo times, 20 views per segment, and 20 reconstructed cardiac phases. For each patient, 8–16 short-axis slices from the base of the heart to the apex and 3 standard LV long-axis views were recorded. Short-axis SSFP images were used for disk-area summation analysis. Three LV long-axis SSFP images (apical 4-chamber, 2-chamber, and long-axis view) were used for the feature tracking analysis.

### 2D speckle tracking analysis

**Fig 1:**
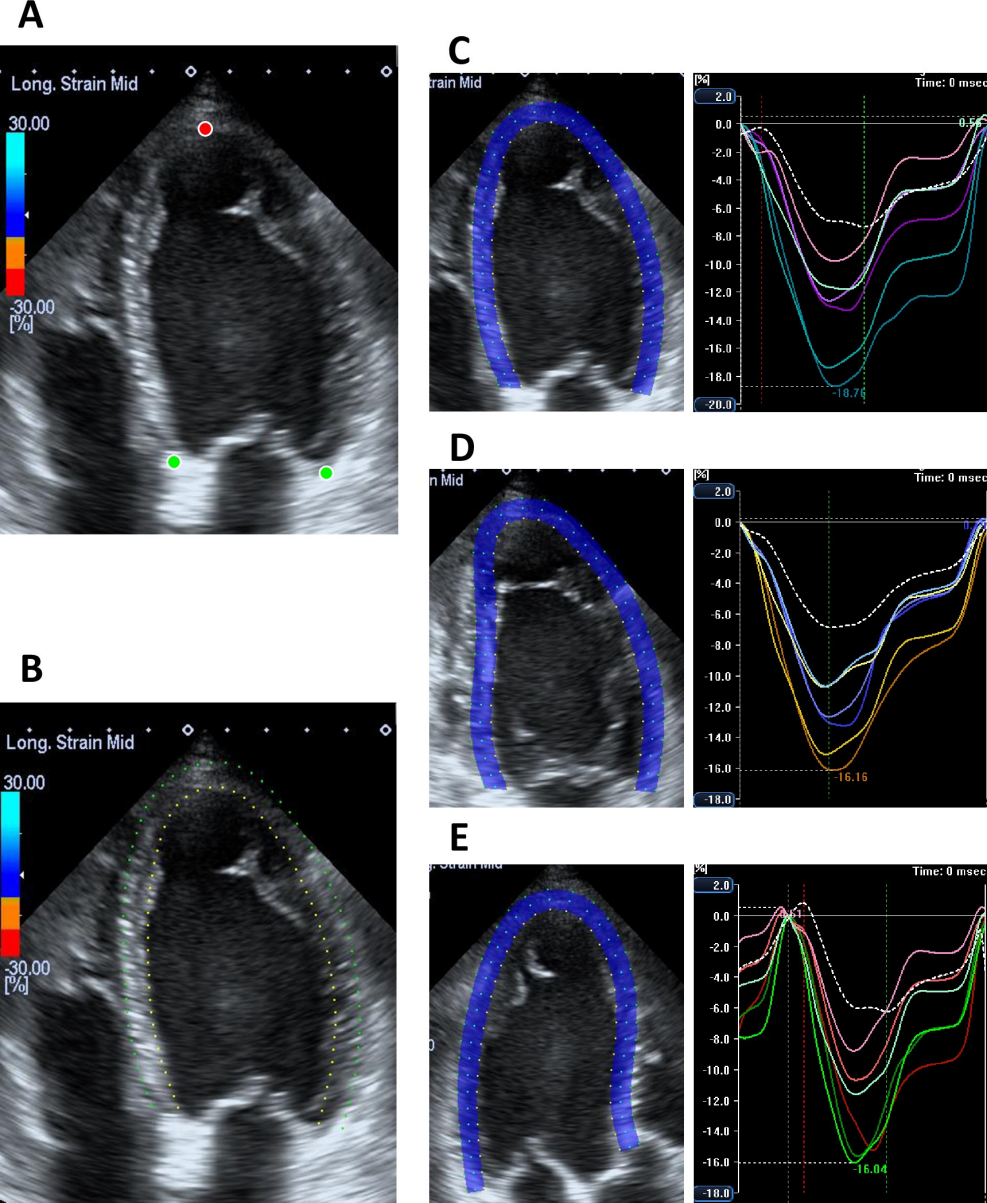
Process of semiautomated layer specific speckle tracking analysis. (A) Apical 4-chamber view at the end-diastole; 3 points (both side of the mitral annulus [green dots] and apex [red dot]) are manually determined. (B) Automatic determination of the endocardial borders (endocardial border [yellow dots] and epicardial border [green dots]. (C) Layer strain analysis on the apical 4-chamber view; blue dots represent mid-myocardial tracking line. The right panel shows segmental strain curve. (D) Layer strain analysis on the apical 2-chamber view. (E) Layer strain analysis on the apical long-axis view.

We used the vendor-dependent prototype software that has an advanced ACT method. First, the examiner identified three specific anatomical landmarks on both sides of the mitral annulus and the apical endocardium at the end-diastolic frame on the apical 4- and 2-chamber views. On the apical long-axis view, the examiner identified the posterior part of the mitral annulus, upper part of the anterior interventricular septum, and the apex (Fig 1A). Subsequently, the new ACT method automatically generated the region of interest (ROI) using a two-step knowledge-based algorithm [7]. The two annulus points were redetected by the classifier based on comparison of the raster scan in the adjacent area where we manually determined annulus points with the pre-learned brightness patterns of the annulus in the annular database. The annular database in 2DE was made using the same method that was previously applied for CMR [8]. Next, the positions of the two redetected annular points and the specified apical points as reference were used for the automatic determination of the final ROI by comparing the input images with the pre-learned contour shape database derived from 100 cases of normal LV shape and 140 abnormal LV shape contours in each apical view (Fig 1B).

We used the same width of ROI in all 3 apical views in each patient, and the width of ROI was adjusted according to the LV wall thickness and usually ranged from 5.1 mm to 6.2 mm. Finally, the software performed speckle tracking analysis on a frame-by-frame basis on the three layers in the ROI (Fig 1C–1E and S1–S3 Movies) and generated time-domain LV volume curves and longitudinal strain profiles for 6 equally divided segments in each apical view, from which the peak strain was determined. The GLS was calculated by averaging the peak longitudinal strains of the 18-segment LV model and expressed as an absolute value. LV volumes and LVEF were calculated using the biplane Simpson’s method with information of each tracking line. To determine the robustness of the software, we did not perform manual editing in any patient. Because the software provides measurements of 3 layers (endocardium, mid-myocardium, and epicardium), we individually presented LV volumes (LV end-diastolic volume, LVEDV; LV end-systolic volume, LVESV), LVEF, and GLS derived from the three layers, and compared these values with the reference values obtained using CMR.

### Standard CMR analysis

The LV volumes were measured from multiple short-axis SSFP images using the disk-area summation method with an analytical software (Segment v2.2, Medviso, Lund, Sweden). LVEF was calculated by LVEDV–LVESV / LVEDV (%).

### CMR feature tracking analysis

The LV volumes, LVEF, and GLS measurements on CMR images were performed using the CMR feature tracking software (2D CPA MR; TomTec Imaging Systems, Unterschleissheim, Germany), which is a vector-based analysis tool based on a hierarchical algorithm. For each of the three apical long-axis SSFP images, the LV endocardial border at the end-systolic frame was semi-automatically determined by a 3-point click (both sides of the mitral annulus and the apex). The software then automatically propagated the contour and followed its features throughout the cardiac cycle to generate LV volume curves from which LVEDV, LVESV, and LVEF were determined. The software also provided segmental peak LS values on the bull’s eye map as well as the GLS.

### Observer variabilities

Intra-observer variabilities of LVEDV, LVESV, LEEF, and GLS were assessed by having the examiner repeat the measurements using the 2DE layer strain analysis, at 2-week interval, in randomly selected 30 patients, and inter-observer variabilities were determined by having a second examiner perform these measurements in the same 30 patients. The intra- and inter-observer variability values were calculated as the absolute differences between the corresponding two measurements in percentages of their mean and intraclass correlation (ICC).

### Statistical analysis

Continuous data are presented as mean ± standard deviation or median and interquartile interval (from the 25^th^ percentile to the 75^th^ percentile) according to data distribution. Categorical data are expressed as numbers or percentages. Repeated measurements of one-way analysis of variance or Friedman’s analysis with post-hoc comparison was performed to compare more than two groups. A linear correlation and Bland-Altman analysis were performed to determine the r value, bias, and 95% confidence interval (CI). Coverage probability (CP) was the probability of the absolute differences falling within the acceptable difference [9]. In accordance with previous studies, the acceptable absolute difference was defined as 30 mL for LVEDV and LVESV, 10% for LVEF, and 4% for GLS. All statistical analyses were performed using commercial software (JMP, version 13.0; SAS Institute, Inc., Cary, NC) and R version 3.4.3. (The R Foundation for Statistical Computing, Vienna, Austria).

### Results

Clinical characteristics in the study population are shown in Table 1. The mean frame rate in the 2DE images was 47 ± 4/sec. Among the 84 study subjects, 2DE image quality was good in 40 (48%), fair in 25 (29%), and poor in 19 (23%). 2DE speckle tracking analysis was not possible in 3 patients because of image storage problem in 1 and electrocardiogram issues in 2 (Feasibility: 96%). The standard CMR method (disk-area summation method) could be performed in all patients (feasibility: 100%). Four patients were excluded from the CMR feature tracking method because of very poor image quality due to artifact from the adjacent structures in 3 and software problem in 1 (feasibility: 95%).

**Table 1:** Clinical characteristics in study population.

Age (years)	68 (56 to 79)
Male/female	49/35
Height (cm)	160 ± 12
Weight (kg)	60 ± 14
BSA (/m^2^)	1.63 ± 0.21
HR (beats/min)	68 ± 15
SBP (mmHg)	129 ± 25
DBP (mmHg)	71 ± 12
Clinical diagnosis	
DCMP	9
ICM	11
Secondary CM	28
VHD	11
HCM	4
PH	5
CPA survivor	3
others	10

Data are expressed as mean ± standard deviation or median (25^th^ to 75^th^ percentile).

BSA, body surface area; CM, cardiomyopathy; CPA, cardiopulmonary arrest; DBP, diastolic blood pressure; DCMP, dilated cardiomyopathy; HCM, hypertrophic cardiomyopathy; HR, heart rate; ICM, ischemic cardiomyopathy; PH, pulmonary hypertension; SBP, systolic blood pressure; VHD, valvular heart disease

### LV volumes

Table 2 depicts LV volumes and LVEF among the four groups in 81 subjects in whom both standard CMR method and 2DE layer strain analysis were possible. The median value of LVEDV using the CMR disk-area summation method was 160 mL. The corresponding value using 2DE endocardial layer tracking was significantly lower than that using CMR (bias: -47 mL, p<0.001) with good correlations (r = 0.87). There were no significant differences in LVEDV between 2DE mid-myocardial layer tracking and CMR (bias: -11 mL, p = 0.091) with good correlation (r = 0.87). In contrast, LVEDV derived using 2DE epicardial tracking was significantly larger than that derived using CMR (bias: 30 mL, p<0.001) with good correlation (r = 0.87). There were no significant differences in LVESV between the standard CMR method and 2DE mid-myocardial tracking.

**Table 2:** Comparison of LV volumes, EF and GLS between new 2D speckle tracking software and CMR disk-area summation method (n = 81).

	CMR	ACTendo	ACTmid	ACTepi	CMR vs. ACTendo	CMR vs. ACTmid	CMR vs. ACTepi
LVEDV	160 (116 to 223)	122 (89 to 167)	156 (119 to 210)	196 (153 to 259)	p<0.001	p = 0.091	p<0.001
r					0.87	0.87	0.87
bias					-47	-11	30
95% LOA					-127 to 34	-87 to 65	-45 to 105
CP					0.33	0.65	0.42
LVESV	99 (62 to 144)	74 (47 to 119)	105 (70 to 157)	141 (98 to 201)	p<0.001	p>0.999	p<0.001
r					0.91	0.91	0.90
bias					-34	-3	33
95% LOA					-109 to 42	-71 to 65	-31 to 97
CP					0.54	0.68	0.40
LVEF	39 (24 to 51)	40 (29 to 47)	34 (25 to 41)	29 (21 to 36)	p = 0.948	p = 0.003	p<0.001
r					0.85	0.84	0.82
bias					1	-4	-9
95% LOA					-16 to 18	-22 to 13	-28 to10
CP					0.77	0.75	0.58

Data are expressed as median (25^th^ to 75^th^ percentile) or mean ± standard deviation.

ACTendo, endocardial tracking with automated contour trace; ACTepi, epicardial tracking with automated contour trace; ACTmid, mid-myocardial tracking with automated contour trace; LOA, limit of agreement; LVEDV, left ventricular end-diastolic volume; LVEF, left ventricular ejection fraction; LVESV, left ventricular end-systolic volume.

Table 3 shows LV volumes, LVEF, and GLS among the four groups in 77 subjects in whom both analyses were possible. The median value of LVEDV using CMR feature tracking was 168 mL, and the corresponding value using 2DE endocardial layer tracking was significantly lower (bias: -54 mL, p<0.001) with good correlations (r = 0.87). Bias of LVEDV between 2DE mid-myocardial layer tracking and CMR decreased but still showed significant differences (bias: -18 mL, p = 0.002). The correlation of LVEDV between the two methods was also good (r = 0.88). In contrast, LVEDV derived using 2DE epicardial tracking was significantly larger than that derived using CMR feature tracking (bias: 23 mL, p<0.001) with good correlation (r = 0.88). The same trend was also observed in LVESV.

**Table 3:** Comparison of LV volumes, EF and GLS between new 2D speckle tracking software and CMR feature tracking method (n = 77).

	CMR	ACTendo	ACTmid	ACTepi	CMR vs. ACTendo	CMR vs. ACTmid	CMR vs. ACTepi
LVEDV	168 (116 to 244)	129 (89 to 169)	164 (119 to 212)	202 (153 to 261)	p<0.001	p = 0.002	p<0.001
r					0.87	0.88	0.88
bias					-54	-18	23
95% LOA					-138 to 30	-95 to 60	-51 to 98
CP					0.26	0.57	0.55
LVESV	102 (70 to 167)	76 (47 to 122)	105 (70 to 161)	142 (98 to 203)	p<0.001	p = 0.140	p<0.001
r					0.91	0.91	0.91
bias					-40	-9	28
95% LOA					-109 to 30	-71 to 53	-32 to 87
CP					0.45	0.70	0.57
LVEF	37 (25 to 43)	39 (29 to 47)	34 (25 to 41)	29 (21 to 36)	p = 0.003	p = 0.001	p<0.001
r					0.90	0.90	0.90
bias					3	-2	-7
95% LOA					-8 to 14	-13 to 8	-17 to 3
CP					0.90	0.94	0.73
GLS	10.4 ± 4.1	10.8 ± 3.7	9.9 ± 3.4	9.0 ± 3.1	P = 0.680	p = 0.270	p<0.001
r					0.86	0.85	0.83
bias					0.4	-0.5	-1.4
95% LOA					-3.8 to 4.6	-4.8 to 3.8	-6.0 to 3.2
CP					0.95	0.92	0.90

Data are expressed as median (25^th^ to 75^th^ percentile) or mean ± standard deviation.

Abbreviations were the same as Table 2.

### LVEF

**Fig 2:**
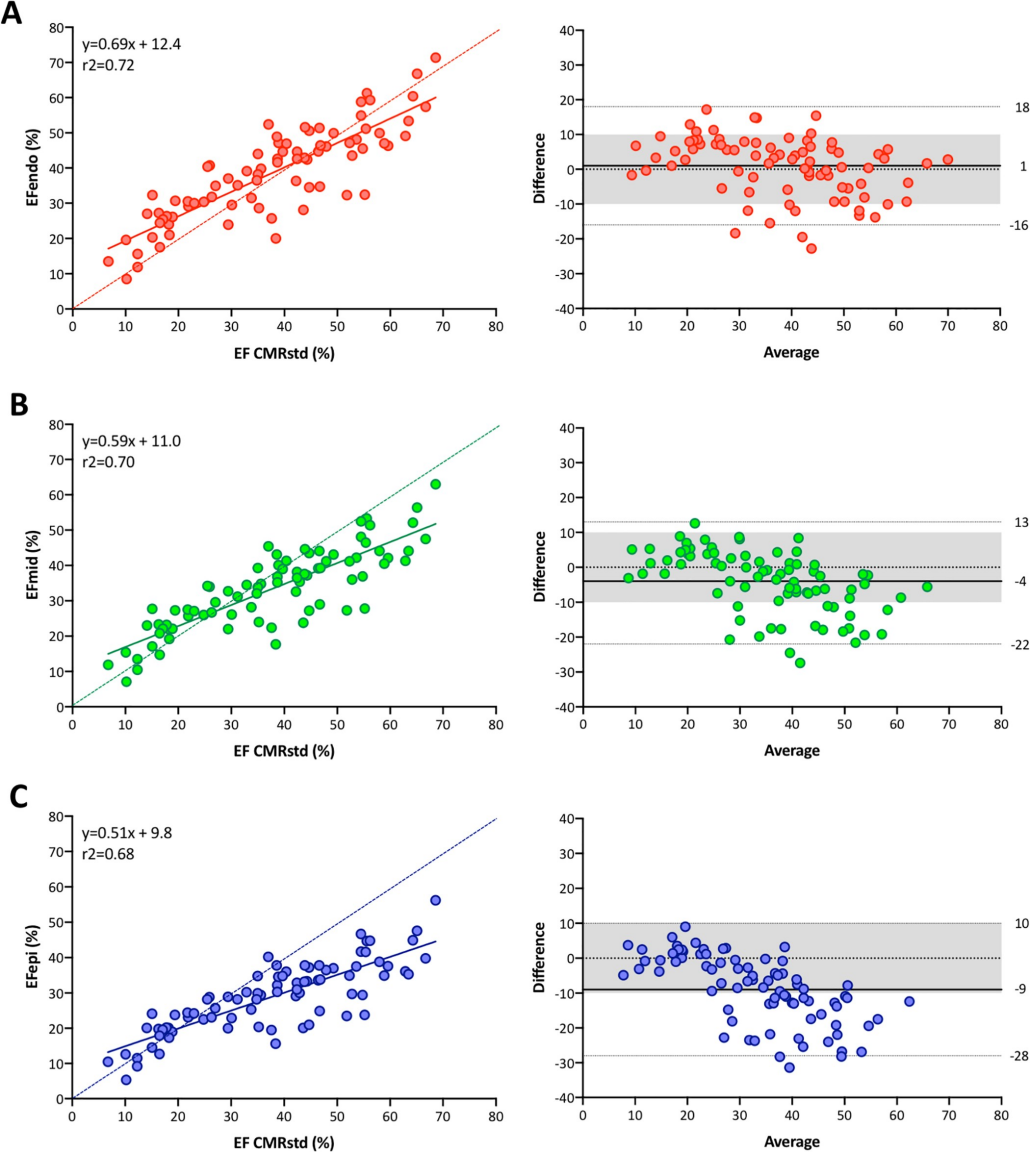
Linear correlation and Bland-Altman analysis regarding LVEF between two methods. (A) Comparison between 2DE endocardial tracking and the standard CMR method. (B) Comparison between 2DE mid-myocardial tracking and the standard CMR method. (C) Comparison between 2DE epicardial tracking and the standard CMR method. A dotted line represents perfect agreement. EF, ejection fraction; CMRstd, standard cardiac magnetic resonance method.

The median value of LVEF using the standard CMR method was 39% (Table 2). The 2DE layer strain analysis determined that LVEF gradually reduced from endocardial layer tracking (median: 40%) to mid-myocardial layer (median: 34%) and epicardial layer (median: 29%) tracking. Post-hoc analysis revealed significant differences in LVEF between CMR and mid-myocardial tracking and between CMR and epicardial tracking. Correlations between the standard CMR method and 2DE speckle tracking ranged from r^2^ = 0.68 to r^2^ = 0.72 (Fig 2). CP was the highest using 2DE endocardial tracking (0.77), followed by mid-myocardial tracking (0.75), and the lowest using epicardial tracking (0.58).

**Fig 3:**
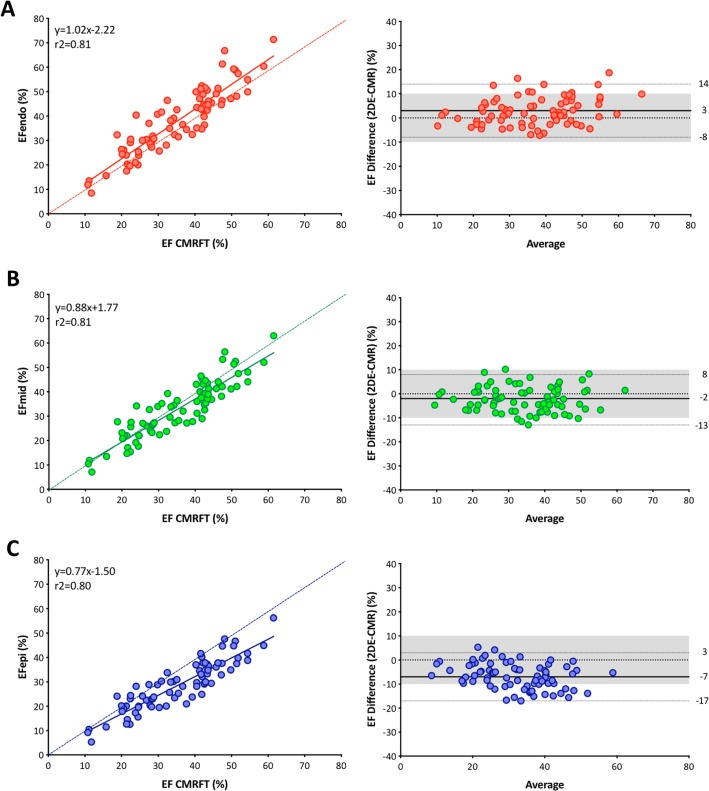
Linear correlation and Bland-Altman analysis regarding LVEF between two methods. (A) Comparison between 2DE endocardial tracking and CMR feature tracking. (B) Comparison between 2DE midmyocardial tracking and CMR feature tracking. (C) Comparison between 2DE epicardial tracking and CMR feature tracking. A dotted line represents perfect agreement. EF, ejection fraction; CMRFT, cardiac magnetic resonance feature tracking.

The median value of LVEF using CMR feature tracking was 37% (Table 3). The 2DE layer strain analysis also determined that LVEF gradually reduced from endocardial layer to epicardial layer tracking. Post-hoc analysis revealed significant differences in LVEF between all paired comparisons. Correlations between CMR feature tracking and 2DE speckle tracking were good (r^2^ = 0.81, 0.80, and 0.80) (Fig 3). CP was the highest using 2DE mid-myocardial tracking (0.94), followed by using endocardial tracking (0.90) and the lowest using epicardial tracking (0.73).

### GLS

**Fig 4:**
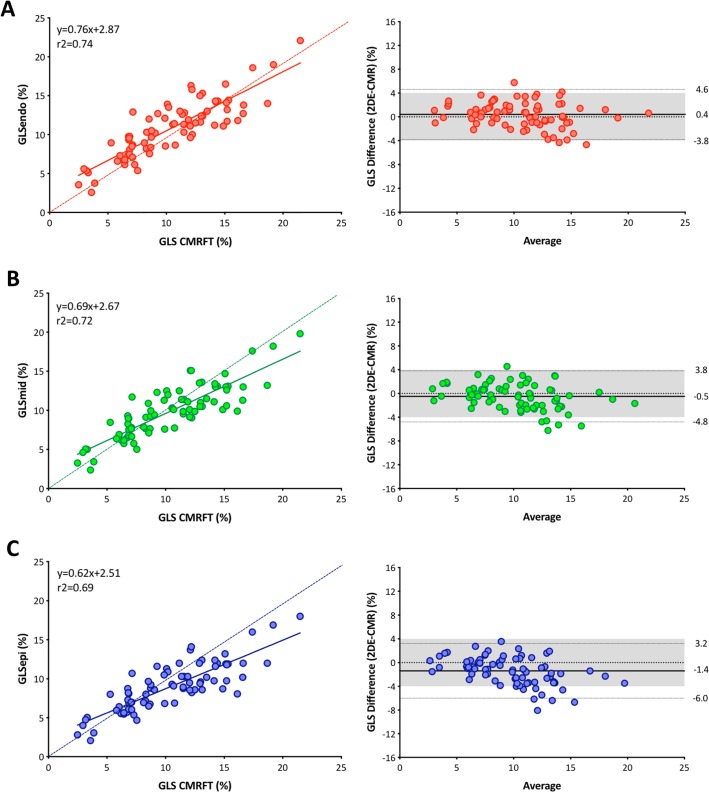
Linear correlation and Bland-Altman analysis regarding GLS between two methods. (A) Comparison between 2DE endocardial tracking and CMR feature tracking. (B) Comparison between 2DE midmyocardial tracking and CMR feature tracking. (C) Comparison between 2DE epicardial tracking and CMR feature tracking. A dotted line represents perfect agreement.

GLS using CMR feature tracking was 10.4 ± 4.1, and the corresponding values using 2DE endocardial, mid-myocardial, and epicardial tracking were 10.8 ± 3.7, 9.9 ± 3.4, and 9.0 ± 3.1, respectively. Post-hoc analysis revealed significant differences between CMR feature tracking and 2DE epicardial tracking (p<0.001). However, no significant differences were noted between CMR feature tracking and 2DE endocardial tracking (p = 0.680) as well as between CMR feature tracking and 2DE mid-myocardial tracking (p = 0.270). Good correlations were noted between CMR feature tracking and 2DE layer speckle tracking (Fig 4). CP was the highest using 2DE endocardial tracking (0.95), followed by using mid-myocardial tracking (0.92) and epicardial tracking (0.90).

### Observer variabilities

Intra and inter-observer variabilities of LV volumes, LVEF, and GLS are presented in Table 4. Intra-observer variabilities with endocardial tracking showed the lowest values, ranging from 1.5% to 3.0%, and gradually increased from endocardial to epicardial tracking. Even though we used epicardial tracking, observer variabilities were still less than 7%. Inter-observer variabilities of LVEF ranged from 3.5% to 5.0%. Inter-observer variabilities of GLS ranged from 4.5% to 6.0%.

**Table 4:** Intra- and inter-observer variability for LV parameters.

		ACTendo	ACTmid	ACTepi	p	p (endo vs. mid)	p (endo vs. epi)	p (mid vs. epi)
Intra-observer variability								
LVEDV	%v	3.0% (1.2 to 7.2%)	3.7% (1.6 to 8.2%)	5.2% (2.8 to 8.6%)	<0.001	1.000	0.083	0.109
	CP	1.0	0.97	0.9				
	ICC	0.986	0.978	0.970				
LVESV	%v	3.0% (1.9 to 9.7%)	5.8% (1.8 to 11.0%)	6.9% (4.0 to 11.7%)	0.006	0.93	0.11	0.42
	CP	1.0	0.97	0.9				
	ICC	0.991	0.978	0.972				
LVEF	%v	1.5% (0.6 to 5.4%)	3.2% (1.3 to 7.2%)	3.9% (2.3 to 5.7%)	0.007	0.014	0.011	0.639
	CP	1.0	1.0	1.0				
	ICC	0.991	0.978	0.977				
GLS	%v	2.2% (1.2 to 5.3%)	2.5% (0.9 to 4.6%)	2.5% (0.8 to 3.9%)	0.1767	-	-	-
	CP	1.0	1.0	1.0				
	ICC	0.989	0.990	0.990				
inter-observer variability								
LVEDV	%v	3.1% (1.9 to 11.5%)	5.1% (2.2 to 11.8%)	7.6% (3.2 to 11.2%)	0.1953	-	-	-
	CP	0.93	0.93	0.80				
	ICC	0.972	0.966	0.949				
LVESV	%v	6.3% (3.3 to 16.7%)	6.8% (2.4 to 14.7%)	9.0% (2.9 to 16.9%)	0.6703	-	-	-
	CP	1.00	0.90	0.83				
	ICC	0.978	0.967	0.951				
LVEF	%v	4.0% (1.6 to 6.9%)	3.5% (1.6 to 7.8%)	5.0% (2.8 to 10.0%)	0.1767	-	-	-
	CP	1.00	1.00	1.00				
	ICC	0.985	0.979	0.966				
GLS	%v	6.0% (3.7 to 10.0%)	4.9% (2.8 to 8.3%)	4.5% (2.2 to 6.3%)	0.3012	-	-	-
	CP	1.00	1.00	1.00				
	ICC	0.967	0.979	0.985				

CP, coverage probability; ICC, intraclass correlation; %v, percent variability. Other abbreviations are the same in Table 2.

## Discussion

The main results of this study can be summarized as follows: (1) Compared to CMR, 2DE endocardial speckle tracking significantly underestimated LV volumes; (2) In contrast, 2DE epicardial tracking significantly overestimated LV volumes and significantly underestimated LVEF; (3) 2DE mid-myocardial tracking provided the lowest bias of LV volumes with high CP values of LVEDV, LVESV, and LVEF; (4) No significant differences in GLS were observed between CMR feature tracking and endocardial tracking as well as between CMR feature tracking and mid-myocardial tracking; (5) Intra- and inter-observer variabilities for LV volumes, LVEF, and GLS were quite low, especially when results from endocardial or mid-myocardial tracking were used.

### Previous studies

There is general agreement that 2DE often underestimates LV volumes because of the foreshortening of the acquired image and geometric assumption. Some previous validation studies on the accuracy of 3DE for LV quantification also described the results from direct comparison of LV volumes and LVEF between 2DE and CMR. All but one study consistently showed 2DE had a larger bias of LV volumes against CMR than 3DE [10–20]. Although the biplane Simpson’s method with 2DE remains the standard method of choice to quantify LV volumes and LVEF in daily clinical practice, manual tracing of the blood and border interface could also result in the underestimation of LV volumes. Because 2DE speckle tracking analysis provides not only GLS but also LV volumes and LVEF, its routine usage is a practical and time saving approach, especially when manual input for the determination of LV endocardial border is minimal.

### Current study

ACT analysis was initially established between the late 1990s and early 2000s for the measurement of LV volumes and LVEF [21]. After several updates to refine the algorithm, the software now has the capability for nearly fully automated contour detection employing knowledge-based workflow and pattern recognition. After the manual registration of three anatomical landmarks, the software corrects the mitral annular points with cluster and randomized tree analysis. Subsequently, the software determines endocardial and epicardial contour, followed by tracking the speckles within the ROI. Because the software does not correct the apical point, only the placement of the apex can affect subsequent LV endocardial border determination, resulting in improved reproducibility of measurements.

LV volume determination using endocardial speckle tracking still showed volume underestimation of 30–55 mL. Although there were no differences in LVEF between endocardial speckle tracking and the standard CMR method, a 3% overestimation of LVEF was observed compared with CMR feature tracking. Although CP values for LVEDV and LVESV were low, CP of LVEF was 0.77 using the standard CMR method or 0.90 using CMR feature tracking, indicating that the bias of LVEF between the two methods was <10% in approximately 80–90% of cases, even though one-fourth of patients had poor image quality. When results from mid-myocardial tracking were used, there was still some underestimation of LVEDV. However, the observed bias (< 20 mL) reached a similar range of bias reported using 3DE with fully automated left chamber quantification software [22, 23]. Compared to CMR feature tracking, mid-myocardial layer speckle tracking analysis for approximating LV volumes and LVEF is recommended because of the lowest bias and highest CP values of LVEF. This finding further supports that the endocardial border on 2DE does not account for the blood volume behind the endocardial trabeculations.

The layer strain analysis revealed transverse gradient of GLS, which was in agreement with previous studies [24, 25]. However, there were no significant differences in GLS between CMR feature tracking and 2DE speckle tracking with endocardial or mid-myocardial layers. The CP values of GLS were also high in all 3 layers. This could be explained by the geometry of longitudinal myocardial deformation. As mitral annular movements of both sides potentially affect longitudinal deformation, GLS in all 3 layers is related to each other unless some part of the myocardial layer shows a strong regional change in curvature [26].

Regarding observer variabilities, the ICC was higher and the coefficient of variation was similar to or lower than those reported in the previous layer specific strain studies [24, 26–28], especially using endocardial or mid-myocardial tracking. This is mainly because manual determination of the apex and width of ROI only affects the ROI contour with this software. These results further support the robustness of this semi-automated layer specific strain analysis software for quantifying global LV mechanics.

### Clinical implications

Quantification always faces two challenges: accuracy and reliability. If a measurement is highly reliable with some limitation regarding accuracy, most clinicians consider it useful. It achieves consistent measurement values on individual images, which is quite useful when conducting a longitudinal study. The excellent reliability of this software suggests that it can detect clinically subtle, but statistically significant changes in LV function during a serial follow-up in observational and interventional studies in a smaller number of subjects compared with manual tracing methods.

### Study limitations

This study has several limitations that should be acknowledged. First, we employed a relatively narrow width of ROI to enhance the tracking quality of the epicardial layer. Thus, our results from epicardial tracking were not solely a reflection of those from the epicardium. Second, after the manual clicking of 3 points in each apical long-axis view, we did not perform manual editing in any case. Although additional manual correction could reduce measurement bias, it may increase observer variabilities. Third, we measured GLS using CMR feature tracking and did not use myocardial tagging. However, CMR feature tracking is increasingly used to quantify heart chamber mechanics [29].

## Conclusions

Using a novel semi-automated 2DE layer specific speckle tracking software, mid-myocardial tracking provided less bias of LV volumes with high CP values of LVEF and GLS against CMR as a reference. Lower observer variability also supports mid-myocardial speckle tracking to quantify LV volumes and function.

## Supporting information

S1 Movie Layer strain analysis on the apical 4-chamber view. (MP4)

S2 Movie Layer strain analysis on the apical 2-chamber view. (MP4)

S3 Movie Layer strain analysis on the apical long-axis view. (MP4)

S1 Dataset Raw dataset. (XLSX)
